# Plasma exosomal miR-30b-5p attenuates neuroinflammation in a rat model of autism spectrum disorder

**DOI:** 10.3389/fpsyt.2025.1630782

**Published:** 2025-10-01

**Authors:** Zhen Zheng, Qiuting Wu, Xingliang Zhang

**Affiliations:** ^1^ Department of Pediatrics, The Third Affiliated Hospital, Sun Yat-sen University, Guangzhou, Guangdong, China; ^2^ Department of Respiratory Medicine, Institute of Pediatrics, Affiliated Shenzhen Children’s Hospital of Shantou University Medical College, Shenzhen, Guangdong, China; ^3^ Institute of Pediatrics, Shenzhen Children’s Hospital, Shenzhen, Guangdong, China

**Keywords:** exosomes, autism spectrum disorder, miR-30b-5p, EGFR, neuroinflammation

## Abstract

**Background:**

There is growing evidence that exosomes play an important role in the pathogenesis of central nervous system diseases, but little is known about the relationship between exosomes and autism spectrum disorder (ASD).

**Methods:**

In this study, a rat model of ASD was generated via prenatal exposure to valproic acid (VPA). Three-chamber social interaction, self-grooming and marble burying tests were utilized for behavioral assessment. The plasma exosomal microRNA (miRNA) expression profiles of VPA-treated rats and sham rats were analyzed. Interleukin-6 (IL-6), tumor necrosis factor-α (TNF-α) and IL-1β levels were measured by ELISA. miR-30b-5p in the brains was assessed by qRT-PCR. Epidermal growth factor receptor (EGFR), p-p38/p38, and CaMKII were assessed by Western blot. In addition, the regulation of miR-30b-5p/EGFR was examined by lateral ventricle injection with miR-30b-5p agomir in VPA-exposed rats.

**Results:**

VPA-exposed rats exhibited ASD-like behaviors. The level of miR-30b-5p was significantly lower in the plasma exosomes and brains of VPA-exposed rats than in those of sham rats. In addition, the levels of inflammatory factors, EGFR, p-p38/p38, and CaMKII were increased in the brains of VPA-exposed rats. Moreover, overexpressing miR-30b-5p ameliorated ASD-like behaviors and decreased the expression of inflammatory factors, EGFR, p-p38/p38, and CaMKII in the brains of VPA-exposed rats.

**Conclusions:**

Our study highlights that plasma exosomal miR-30b-5p attenuates neuroinflammation in a rat model of ASD by modulating EGFR through the MAPK signaling pathway and calcium signaling pathway. This study provides novel perspectives on plasma exosomal miR-30b-5p, which could be considered a potential therapeutic target for the treatment of ASD in the clinic.

## 1 Introduction

Autism spectrum disorder (ASD) is a neurodevelopmental disorder characterized by impaired social interaction, communication deficits and stereotypical or repetitive behavior. In the U.S., one in every 36 children suffers from ASD (1). ASD occurs early and lasts for a lifetime, affecting the quality of life of people with ASD and placing an economic burden on their families (2). ASD is recognized as a major global public health problem (3). However, the mechanism underlying ASD is unclear. Moreover, there is no specific effective treatment for ASD.

Inflammation has been reported to participate in the pathogenesis of ASD (4–8). Previous studies have shown elevated levels of inflammatory factors such as interleukin-6 (IL-6), IL-1β and tumor necrosis factor-α (TNF-α) in the blood of children with ASD (4, 6, 8). In addition, the levels of inflammatory factors, including IL-6, TNF, interferon (IFN)-γ, and IL-2 levels, are increased in the brains of ASD model (5, 7), which suggests that inflammation might be closely coupled with ASD pathogenesis. Previous studies have reported that modulating neuroinflammation can ameliorate autism-related behaviors in ASD model (9, 10). Thus, targeting the modulation of neuroinflammation may constitute a new strategy for treating ASD.

Recently, exosomal microRNAs (miRNAs) have been treated as biomarkers and potential treatments for a variety of central nervous system diseases (11–13). Exosomes are extracellular vesicles between 30 and 150 nm in diameter (14). These particles have phospholipid bilayers that can transport metabolites, lipids, proteins, mRNAs, miRNAs, and long noncoding RNAs (15, 16). In the central nervous system, exosomes are produced by all types of cells in the brain, including neurons, oligodendrocytes, astrocytes, and microglia (17). Exosomes from brain cells composed of intracranial substances are delivered to the peripheral blood by crossing the blood-brain barrier (18, 19). Thus, miRNAs in plasma exosomes might reflect changes in the brain (20). However, miRNAs derived from exosomes in ASD remain underexplored.

miRNAs are small RNA molecules that regulate gene expression and bind to targets in the noncoding regions of mRNA (21). miRNAs play vital roles in neurogenesis, neuronal differentiation, and synaptogenesis (22, 23). miR-30b-5p has beneficial roles in attenuating apoptosis following epilepsy (24). Moreover, miR-30b-5p can be detected in large quantities in the plasma exosomes of patients with Alzheimer’s disease (25). However, the specific role of plasma exosomal miR-30b-5p in ASD has not been elucidated.

In this study, we aimed to characterize the expression profile of miRNAs in the plasma exosomes and further examine the mechanism of plasma exosomal miR-30b-5p in a rat model of ASD. Our results indicate that plasma exosomal miR-30b-5p could be considered a potential therapeutic target for the treatment of ASD in the clinic.

## 2 Materials and methods

### 2.1 Animals

Sprague-Dawley rats were purchased from the Animal Experiment Center of Southern Medical University. The experiments were performed in accordance with the National Institutes of Health Guidelines for the Care and Use of Laboratory Animals, and all the animal protocols were approved by local authorities.

### 2.2 Drugs and treatments

To obtain pregnant animals, virgin females and males that were 10–12 weeks old were placed together overnight, and the morning of sperm discovery was specified as the first day of pregnancy. To induce ASD-like behaviors in offspring, pregnant rats were intraperitoneally injected with 500 mg/kg valproic acid (VPA, Sigma, P4543) at embryonic day 12.5 (26). VPA was dissolved in saline at a concentration of 250 mg/ml. A total of 6 pregnant dams were administered VPA, whereas three pregnant dams received an injection of saline at the same time. The offspring of saline-injected dams served as the sham group. The offspring of VPA-injected dams were viewed as the VPA-exposed group. After weaning at postnatal day 21 (PND21), male offspring were housed independently with 4–5 rats per cage and investigated in this study. The male offspring were subjected to behavioral tests at the specified time points, with ten male rats included in each group.

### 2.3 Behavioral tests

Throughout the entire study and subsequent data analysis, researchers were kept blind to the group assignments. To eliminate the interference of circadian rhythms, behavioral tests were conducted at the same fixed times each day. The following exclusion criteria were applied: rats were excluded from the analysis in cases of mortality, a body weight loss exceeding 20% of the initial weight, or failure to complete the behavioral tests (e.g., due to persistent immobility or aggressive behavior).

#### 2.3.1 Three-chamber test

To assess the ASD phenotype, a three-chamber instrument was used for the social behavior test. In the sociability test, a stranger rat (strange 1) was placed in the left chamber, while an empty cage was placed in the right chamber. The experimental rats were placed in the middle chamber and tested for 10 minutes. Then, in the social preference test, the empty cage was removed, and another unfamiliar normal rat was placed in the left chamber as the unfamiliar rat. The strange 1 rat in the right chamber was the familiar rat. The experimental rat was placed in the central chamber and allowed to explore the familiar and unfamiliar rats for another 10 minutes. The time spent in each chamber was recorded. The sociability index and social preference index were calculated using the following formulas:


Sociability index=total time in the stranger chamber/total time in the empty chamber

Social preference index = total time in the unfamiliar chamber/total time in the familiar chamber

#### 2.3.2 Self-grooming test

Self-grooming was conducted to evaluate repetitive behaviors. Each rat was placed in an empty cage and observed for 10 minutes. The accumulated self-grooming time was recorded for 10 minutes. Grooming was defined as rubbing two forelimbs against the head, face, or body.

#### 2.3.3 Marble burying test

The marble burying test was performed to evaluate anxiety behaviors. The rats were placed separately in a cage with 5 cm deep clean bedding. Twenty glass marbles were placed on top of the bedding. The rats were tested for 30 minutes. Marbles submerged in bedding to at least two-thirds of their depth were counted.

### 2.4 Blood collection

The rats were anesthetized at PND 60. The blood was collected and stored at 4°C for a short time and then centrifuged at 3,000 g at 4°C for 10 minutes. Fourteen samples (seven from VPA-exposed rats and seven from sham rats) were used for exosome isolation.

### 2.5 Isolation and detection of exosomes

Exosomes were separated from plasma by differential centrifugation (2,000 g for 30 min, 10,000 g for 45 min, and 100,000 g for 70 min) at 4°C and washed with PBS. Then, the supernatant was removed, and the exosomes were resuspended in PBS.

Exosomes were observed via transmission electron microscope (TEM). Nanoparticle tracking analysis (NTA) was used to determine the size distribution of the plasma exosomes. Proteins were extracted from the plasma exosomes of both groups. Typical markers of exosomes were detected by Western blotting. The primary antibodies used included Alix (Abcam, ab275377, 1:1,000), CD81 (Abcam, ab109201, 1:1,000), and TSG101 (Abcam, ab125011, 1:3,000).

### 2.6 miRNA sequencing and subsequent bioinformatics analyses

Exosomes were isolated from fourteen rats, among which, exosomal miRNA extracted from six rats (Sham: VPA = 1:1) was divided into two aliquots per rat: one for miRNA sequencing and the other for subsequent RT-qPCR validation. Exosomal miRNA extracted from an additional six rats (randomly selected from the remaining eight rats not used for sequencing, with Sham: VPA = 1:1) was also used for subsequent RT-qPCR validation.

miRNA sequencing and analysis were conducted by OE Biotech Co., Ltd. (Shanghai, China). The miRBase v.21 database was used to analyze the known miRNAs in different samples. miRDeep2 was used to analyze unannotated small RNAs to predict novel miRNAs. The corresponding miRNA star sequence was obtained according to the hairpin structure of the pre-miRNA and the miRbase database.

Kyoto Encyclopedia of Genes and Genomes (KEGG) pathway enrichment analyses of differentially expressed miRNA target genes were performed using R according to the hypergeometric distribution.

### 2.7 qRT-PCR analysis

Quantification analysis was carried out using a two-step reaction process. The primers of the selected miRNAs are shown in **Table 1**. Each sample was analyzed in triplicate. The expression levels of the miRNAs were calculated using the 2^-ΔΔCt^ method.

**Table 1:** Primer sequences used in this study.

miRNA	Primer type	Primer Sequence (5’-3’)
rno-miR-145-5p	Forward	AGTTTTCCCAGGAATCCCTAA
rno-miR-199a-3p	Forward	AGTAGTCTGCACATTGGTTAAA
rno-miR-125b-5p	Forward	CTGAGACCCTAACTTGTGAAA
rno-miR-486	Forward	TACTGAGCTGCCCCGAGAA
rno-miR-30b-5p	Forward	GTAAACATCCTACACTCAGCT
rno-miR-25-3p	Forward	TTGCACTTGTCTCGGTCTGA
rno-miR-148b-3p	Forward	AGTGCATCACAGAACTTTGTAA

### 2.8 Intracerebroventricular infusion of miR-30b-5p

At PND 60, the miR-30b-5p agomir and its negative control (NC) were dissolved and mixed according to the manufacturer’s instructions (RiboBio, China). After the rats were anesthetized with pentobarbital sodium (40 mg/kg), the skulls were exposed. The miR-30b-5p agomir (5 nM) or its NC was injected into the lateral ventricles of VPA-exposed rats using a Hamilton syringe. A Hamilton syringe was inserted at 0.8 mm posterior to the bregma, 1.8 mm lateral to the sagittal suture, and 3.6 mm beneath the cortical surface (27). A 5 μL volume of miR-30b-5p agomir or its NC was injected into the lateral ventricles of the rat at a rate of 1 μL/min for 5 minutes (28). The rats were divided into the VPA+ miR-30b-5p agomir and VPA+ miR-30b-5p agomir NC groups. There were ten male rats in each group.

### 2.9 Behavioral tests and tissue preparation

On day 3 after the miR-30b-5p agomir or NC was infused, the rats were transferred for behavioral tests. After the behavioral tests, the rats were deeply anesthetized. For Western blotting, qRT-PCR and ELISA analyses, the brains were rapidly removed and stored at -80°C.

### 2.10 Western blotting

The specific steps of the Western blot analysis were performed as previously described (29). The membranes were incubated with primary antibodies against epidermal growth factor receptor (EGFR) (Abcam, ab32562, 1:5000), p38 (CST, 8690T, 1:1000), p-p38 (CST, 4511T, 1:1000), CaMKII (Abcam, ab52476, 1:3000) and Gapdh (CST, 2118s, 1:1,000), and then subsequently washed and incubated with a secondary antibody (1:5,000, Boster, BA1054). Finally, the protein bands were observed via enhanced chemiluminescence.

### 2.11 ELISA

The expression levels of IL-6, TNF-α, and IL-1β in the brain were evaluated using rat ELISA kits (Ek-Bioscience, Shanghai, China).

### 2.12 Statistical analysis

All the statistical analyses were conducted in GraphPad Prism 10.5.0. Quantitative data are presented as mean ± standard deviation (SD). An unpaired two-tailed Student’s t-test was employed for comparisons between two groups following confirmation of data normality and homogeneity of variances. If the assumptions of normality or equal variances were violated, a non-parametric rank-sum test was utilized instead. For categorical data of marble burying test, data are expressed as median with interquartile range (IQR), and the Mann-Whitney U test was applied to assess differences between two groups. *p* < 0.05 was considered significant.

## 3 Results

### 3.1 VPA-exposed rats exhibited ASD-like behaviors

**Figure 1:**
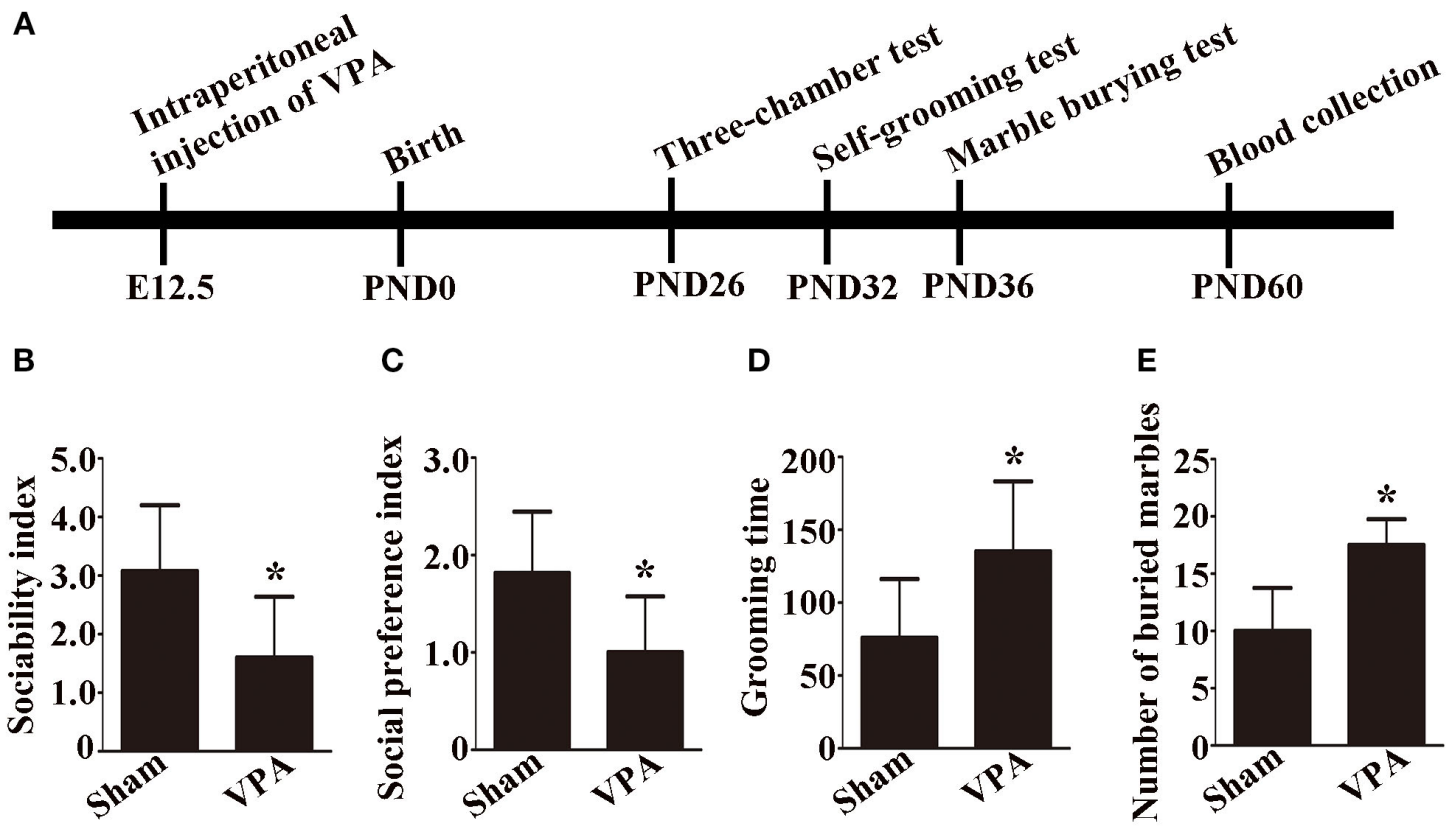
Experimental procedural timeline, social behavior, and repetitive and anxiety behaviors of VPA-exposed rats. **(A)** Experimental procedural timeline. **(B)** Sociability index; n = 6/group. **(C)** Social preference index; n = 6/group. **(D)** Grooming time; n = 6/group. **(B-D)** Data are expressed as mean ± SD. **(E)** Number of buried marbles in the marble burying test; n = 8/group; Data are expressed as median with IQR. *p < 0.05 for the VPA group versus the sham group.

The experimental procedural timeline is shown in **Figure 1A**. Pregnant rats were injected with 500 mg/kg VPA or saline intraperitoneally on embryonic day 12.5. The date of birth was PND 0. The three-chamber test was performed on PND 26. A self-grooming test was conducted on PND 32. The marble burying test was performed on PND 36. The rats were anesthetized, and peripheral blood was collected on PND 60.

In the sociability test, the sociability index was significantly lower in VPA-exposed rats than in sham rats (**Figure 1B**, p < 0.05). In the social preference test, the social preference index of the VPA-exposed rats was significantly lower than that of the sham rats (**Figure 1C**, p < 0.05).

A self-grooming test was used to evaluate repetitive behaviors, and a marble burying test was used to assess anxiety behaviors in the rats. In the present study, the results of the self-grooming test revealed that the VPA-exposed rats spent more time on self-grooming than the sham rats did (**Figure 1D**, p < 0.05). Compared with sham rats, VPA-exposed rats buried more marbles in the marble burying test (**Figure 1E**, p < 0.05). The results demonstrated that a rat model of ASD was successfully established.

### 3.2 Detection of exosomes and differential expression of miRNAs in the plasma exosomes of VPA-exposed rats

**Figure 2:**
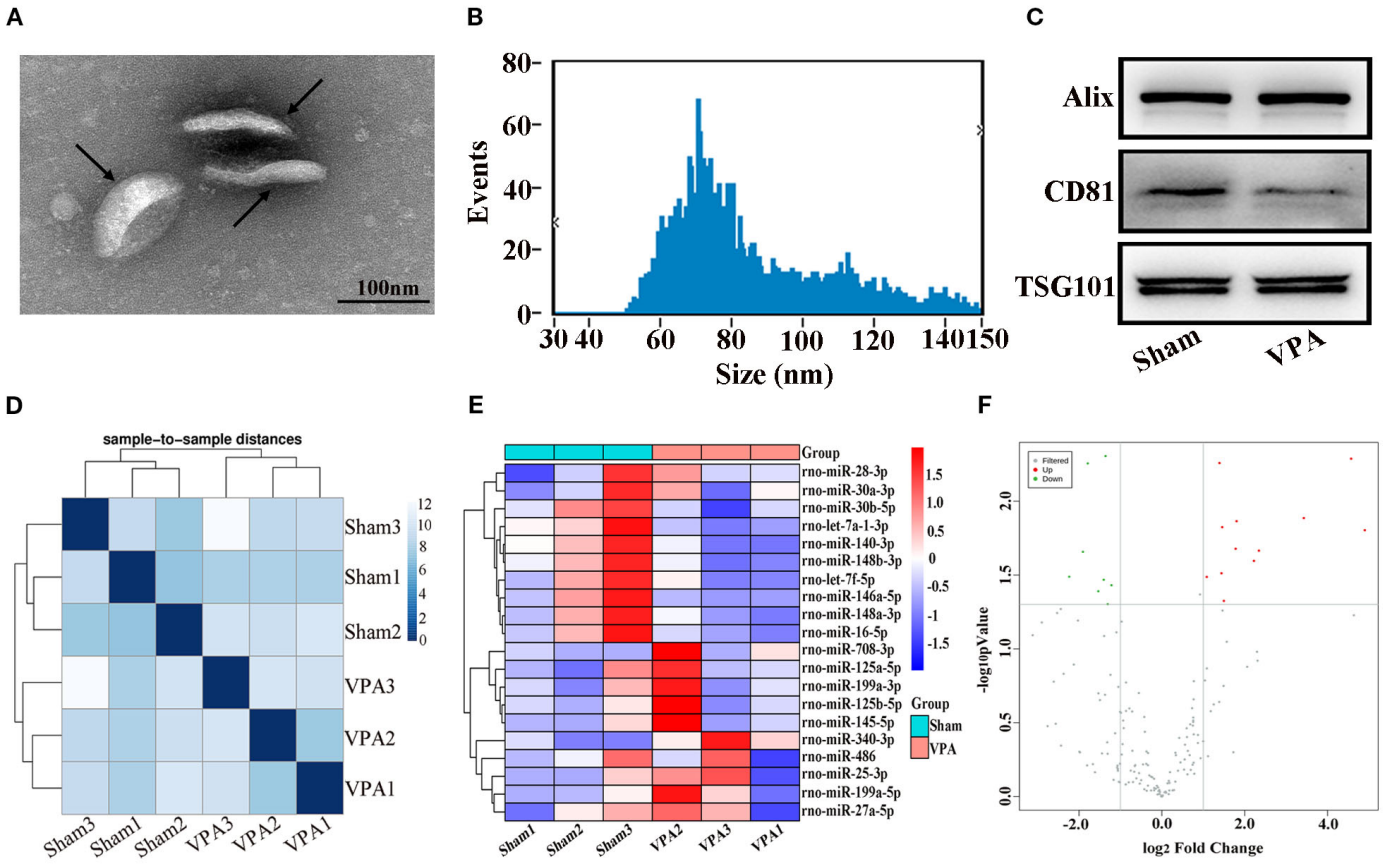
Identification of exosomes and correlation of samples and differentially expressed miRNAs in the plasma exosomes of VPA-exposed rats. **(A)** Transmission electron microscope image of exosomes. Black arrows show typical exosomes. **(B)** Results of the nanoparticle tracking analysis of the exosomes. **(C)** Western blot analysis of exosomes markers Alix, CD81, and TSG101. **(D)** The heatmap shows sample-to-sample distances, and samples with high similarity were preferentially clustered together. **(E)** Heat plot of differentially expressed miRNAs. **(F)** Volcano plot of differentially expressed miRNAs.

TEM analysis revealed that the exosomes were irregular spheres with clear and relatively intact membranes (**Figure 2A**). The NTA results revealed that the diameter of the exosomes varied from 30 to 150 nm, with a median of 77.25 nm, indicating a wide range of exosomes (**Figure 2B**). Western blot analysis revealed that the exosomes of VPA-exposed rats and sham rats expressed typical exosome markers, such as Alix, CD81, and TSG101 (**Figure 2C**). Briefly, we successfully obtained plasma exosomes.

The miRNAs from the plasma exosomes of three VPA-exposed rats and three sham rats were sequenced. The miRNA-seq reads of each sample are shown in **Table 2**. The heatmap shows sample-to-sample distances and preferentially shows samples with high similarity clustering (**Figure 2D**). Compared with the miRNA expressions in sham rats, we found 20 significantly differentially expressed miRNAs in the plasma exosomes of VPA-exposed rats, of which 12 were upregulated and 8 were downregulated (**Table 3**). We constructed a heat plot of 20 differentially expressed miRNAs to display the recognizable miRNA expression profiles of the samples (**Figure 2E**). A volcano plot was constructed based on the basis of *p* < 0.05 and ∣log_2_FC∣ > 1.0 (**Figure 2F**).

**Table 2:** miRNA-seq read statistics.

Sample	Raw reads	miRNA number
Sham 1	9899987	172
Sham 2	17133796	187
Sham 3	13993630	223
VPA 1	9672102	163
VPA 2	8986661	170
VPA 3	11478690	132

**Table 3:** Top 20 differentially expressed miRNAs.

miRNA_id	log2FoldChange	pValue	Regulation	Sequence	Length
rno-miR-148b-3p	-1.35874	0.004947	Down	TCAGTGCATCACAGAACTTTGT	22
rno-miR-340-3p	4.568588	0.005159	Up	TCCGTCTCAGTTACTTTATAGCC	23
rno-miR-28-3p	1.390985	0.00552	Up	CACTAGATTGTGAGCTCCTGGA	22
rno-miR-140-3p	-1.78868	0.00555	Down	TACCACAGGGTAGAACCACGG	21
rno-miR-145-5p	3.426898	0.013008	Up	GTCCAGTTTTCCCAGGAATCCCT	23
rno-miR-125a-5p	1.803952	0.013662	Up	TCCCTGAGACCCTTTAACCTGTGA	24
rno-miR-27a-5p	1.455813	0.014997	Up	AGGGCTTAGCTGCTTGTGAGCA	22
rno-miR-708-3p	4.899731	0.015724	Up	CAACTAGACTGTGAGCTTCTAG	22
rno-miR-199a-3p	1.781018	0.02099	Up	ACAGTAGTCTGCACATTGGTTA	22
rno-miR-199a-5p	2.341471	0.021627	Up	CCCAGTGTTCAGACTACCTGTTC	23
rno-miR-30b-5p	-1.90618	0.022018	Down	TGTAAACATCCTACACTCAGCT	22
rno-miR-125b-5p	2.221926	0.025369	Up	TCCCTGAGACCCTAACTTGTGA	22
rno-miR-25-3p	1.43984	0.030755	Up	CATTGCACTTGTCTCGGTCTGA	22
rno-let-7a-1-3p>rno-let-7c-2-3p	-2.23677	0.032477	Down	CTATACAATCTACTGTCTTTCC	22
rno-miR-30a-3p	1.083535	0.032548	Up	CTTTCAGTCGGATGTTTGCAGC	22
rno-miR-16-5p	-1.40262	0.034033	Down	TAGCAGCACGTAAATATTGGCG	22
rno-let-7f-5p	-1.2152	0.037065	Down	TGAGGTAGTAGATTGTATAGTT	22
rno-miR-148a-3p	-1.53512	0.040714	Down	TCAGTGCACTACAGAACTTTG	21
rno-miR-486	1.496618	0.047305	Up	TCCTGTACTGAGCTGCCCCGAG	22
rno-miR-146a-5p	-1.30662	0.049789	Down	TGAGAACTGAATTCCATGGGTT	22

### 3.3 Verification of the accuracy of the miRNA sequencing results by qRT-PCR

**Figure 3:**
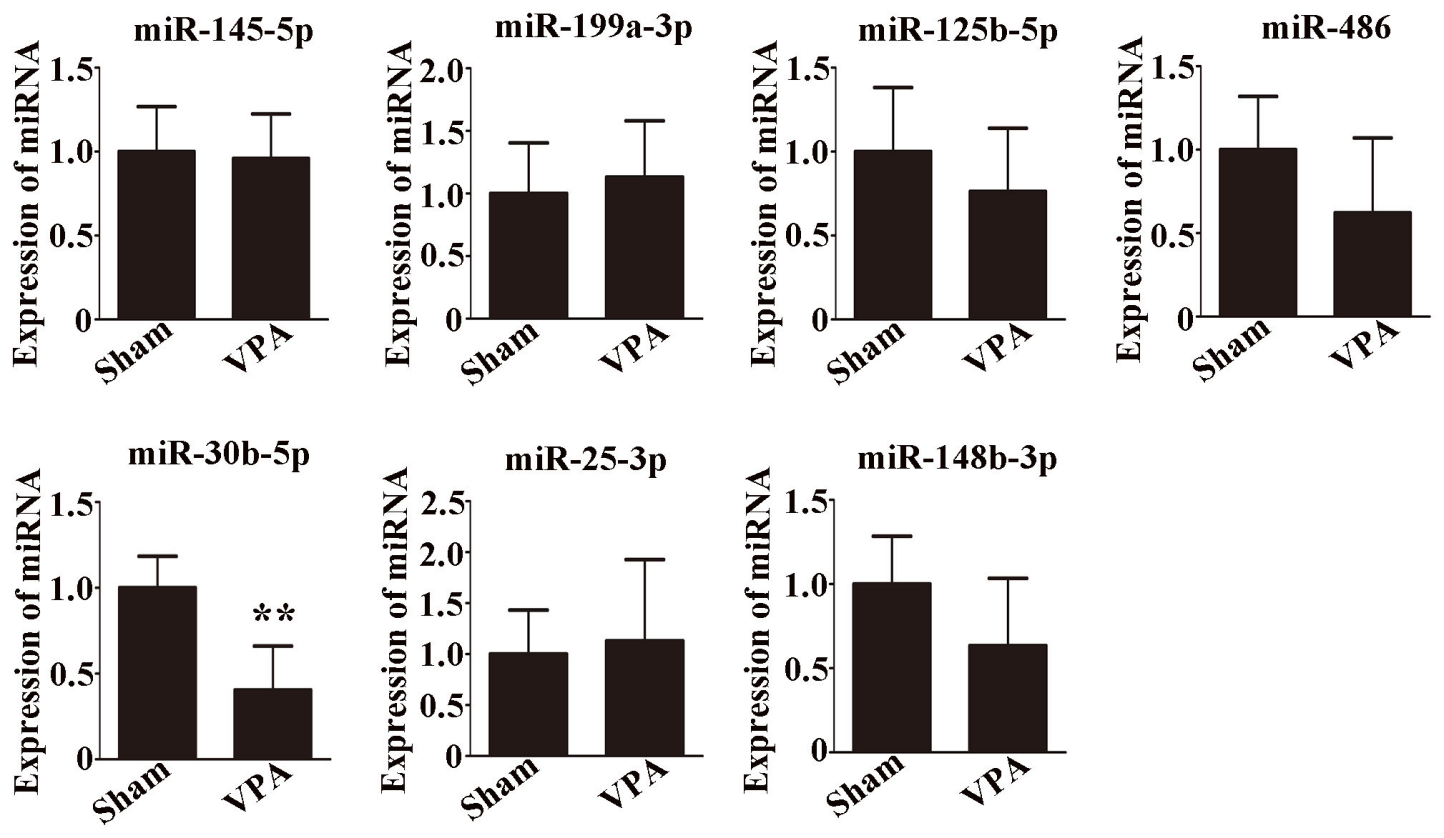
Validation of seven selected miRNAs by qRT-PCR. n = 6/group and each experiment was performed with 3 technical replicates. The data are expressed as the mean ± SD. **p < 0.01 for the VPA group versus the sham group.

Seven differentially expressed miRNAs were chosen for verification. The expression levels of these miRNAs were compared by qRT-PCR and are shown in **Figure 3**. There was a significant difference in the expression of miR-30b-5p among all the selected miRNAs (**Figure 3**, p < 0.01). Moreover, the trend of miR-30b-5p expression was consistent with the miRNA sequencing result.

### 3.4 KEGG pathway analysis of differentially expressed miRNA target genes

**Figure 4:**
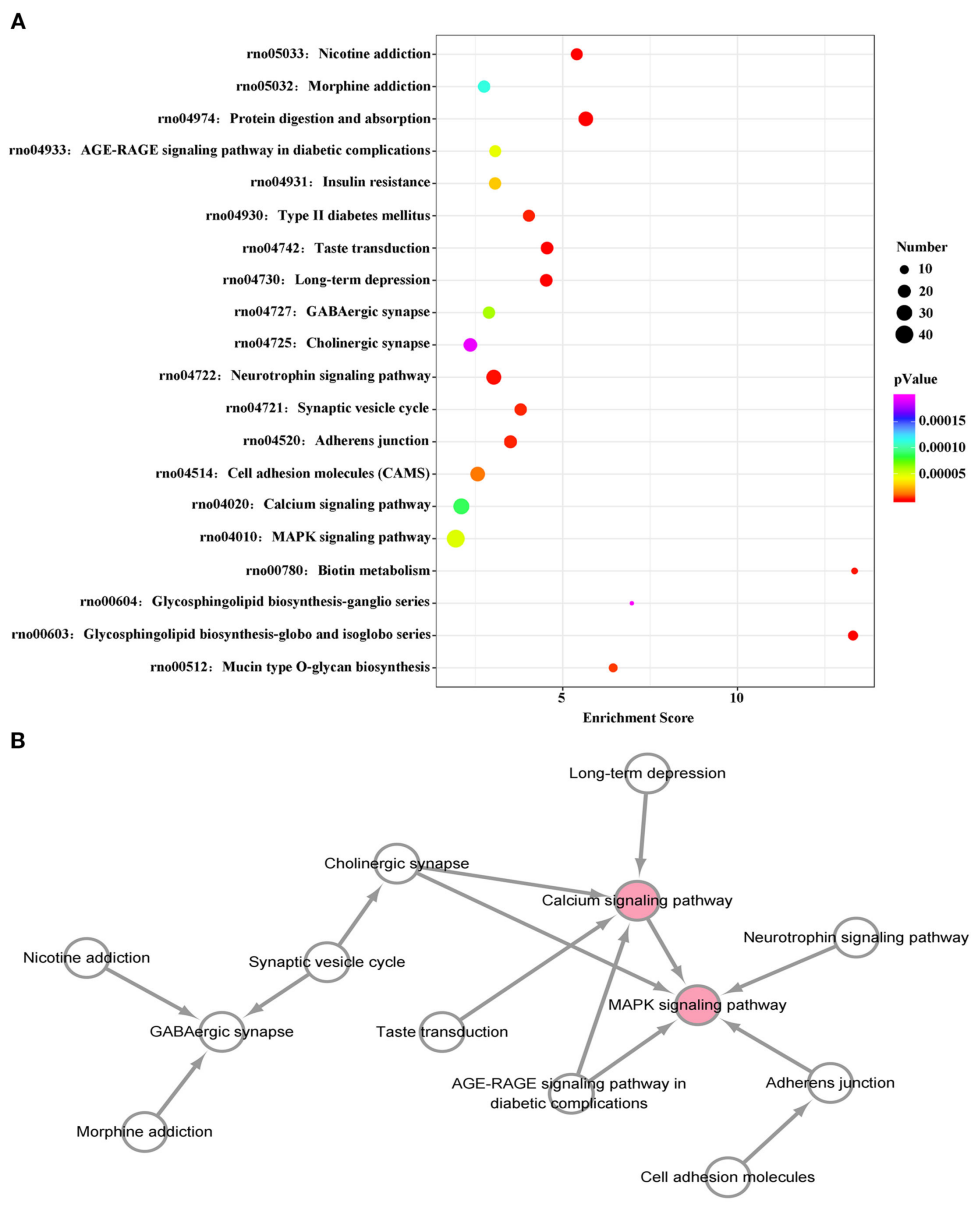
KEGG pathway analysis of differentially expressed miRNAs. **(A)** KEGG enrichment analysis shows the top 20 KEGG pathways. The size of dots represents number of genes, while the color of the dots indicates the *p* value. The horizontal axis represents the enrichment score. **(B)** The interaction network diagram of the top 20 KEGG pathways. Pink spots represent core pathways.

A KEGG pathway dot plot was used to represent the top 20 enrichment pathways. KEGG pathway analysis revealed the pathways of differentially expressed miRNAs in the plasma exosomes of VPA-exposed rats (**Figure 4A**). The top 20 pathways were as follows: mucin type O-glycan biosynthesis, glycosphingolipid biosynthesis-globo and isoglobo series, glycosphingolipid biosynthesis-ganglio series, biotin metabolism, the MAPK signaling pathway, calcium signaling pathway, cell adhesion molecules, adherens junction, the synaptic vesicle cycle, the neurotrophin signaling pathway, cholinergic synapse, GABAergic synapse, long-term depression, taste transduction, type II diabetes mellitus, insulin resistance, the AGE-RAGE signaling pathway in diabetic complications, protein digestion and absorption, morphine addiction, and nicotine addiction.

We constructed a pathway relationship network of the top 20 significant pathways of the differentially expressed miRNAs (**Figure 4B**), which indicated that the MAPK signaling pathway and calcium signaling pathway were among the most central pathways in the VPA-exposed rats.

### 3.5 Exosomal miR-30b-5p has an effect on neuroinflammation by modulating EGFR through the MAPK signaling pathway and calcium signaling pathway

**Figure 5:**
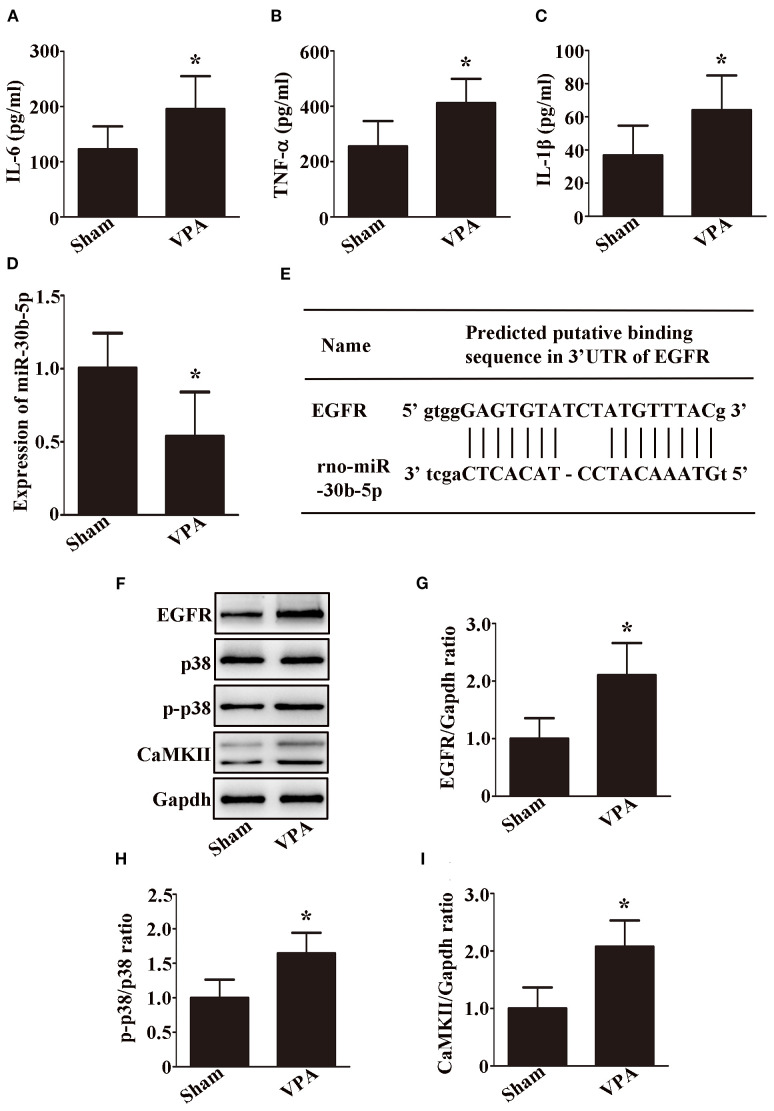
Exosomal miR-30b-5p has an effect on neuroinflammation by modulating EGFR through the MAPK signaling pathway and calcium signaling pathway. **(A–C)** IL-6, TNF-α, and IL-1β levels in the brain were examined by ELISA; n = 6/group and each experiment was performed with 3 technical replicates. **(D)** Expression of miR-30b-5p in the brain was examined by qRT-PCR; n = 6/group and each experiment was performed with 3 technical replicates. **(E)** Diagram showing the direct targeting of miR-30b-5p on the UTR of EGFR. **(F–I)** Representative images and quantifications of EGFR, p38, p-p38, and CaMKII expressions in the brain; n = 3/group and each experiment was performed with 3 technical replicates. The data are expressed as the mean ± SD. *p < 0.05 for the VPA group versus the sham group.

We further examined the effect of miR-30b-5p on neuroinflammation *in vivo*. As shown in **Figures 5A–D**, compared with those of sham rats, IL-6, TNF-α, and IL-1β were upregulated, whereas miR-30b-5p was downregulated in the brains of VPA-exposed rats (*p* < 0.05). Using bioinformatic analysis, we speculated that EGFR could be a potential target gene of miR-30b-5p (**Figure 5E**). Western blot analysis revealed that the expression of EGFR was greater in the brains of VPA-exposed rats than in those of sham rats (**Figures 5F, G**, p < 0.05). Moreover, we examined whether the MAPK signaling pathway and calcium signaling pathway are involved in VPA-exposed rats. Compared with the sham rats, the levels of p-p38/p38, and CaMKII were increased in the brains of VPA-exposed rats (**Figures 5F, H, I**, p < 0.05). These results indicate that exosomal miR-30b-5p has an effect on neuroinflammation by modulating EGFR through the MAPK signaling pathway and calcium signaling pathway.

### 3.6 Overexpression of miR-30b-5p ameliorates the ASD-like behaviors by modulating EGFR through the MAPK signaling pathway and calcium signaling pathway

**Figure 6:**
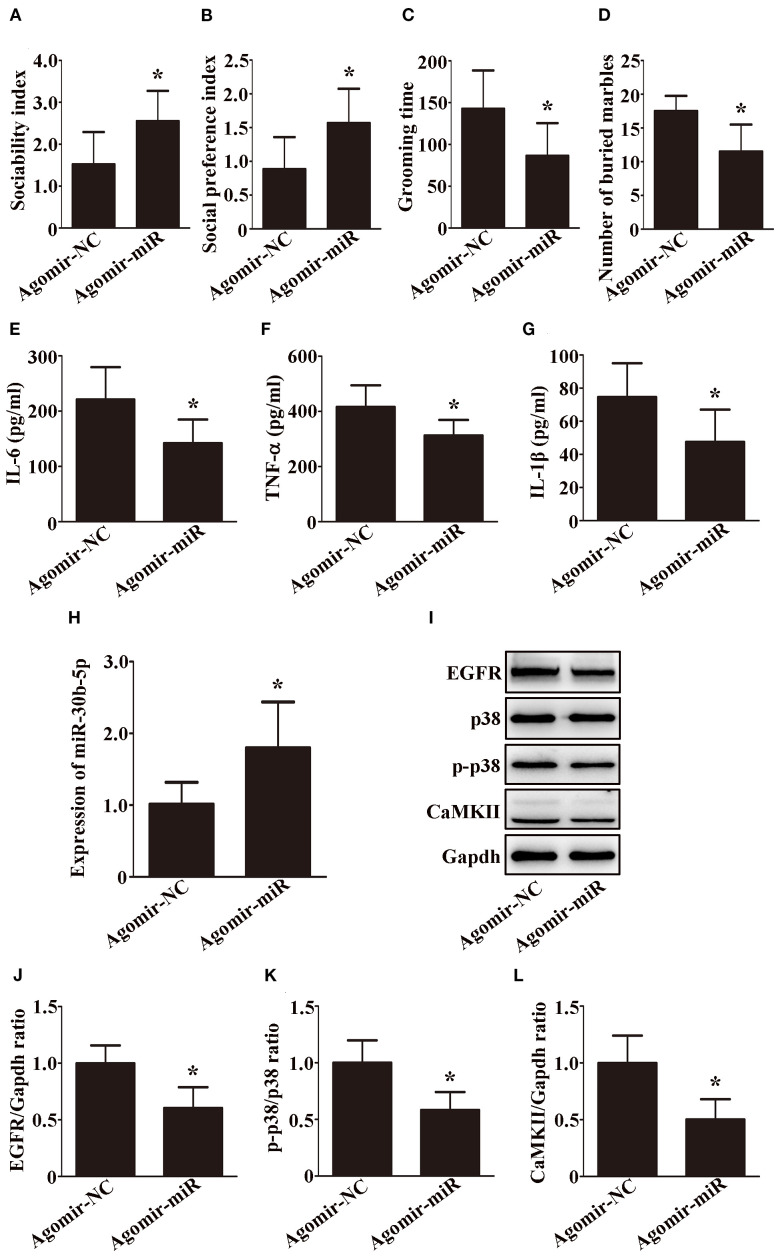
The overexpression of miR-30b-5p ameliorates ASD-like behaviors by modulating EGFR through the MAPK signaling pathway and calcium signaling pathway. **(A)** Sociability index; n = 6/group. **(B)** Social preference index; n = 6/group. **(C)** Grooming time; n = 6/group. **(D)** Number of buried marbles in the marble burying test; n = 8/group; Data are expressed as median with IQR. **(E–G)** IL-6, TNF-α, and IL-1β levels in the brain were examined by ELISA; n = 6/group and each experiment was performed with 3 technical replicates. **(H)** Expression of miR-30b-5p in the brain was examined by qRT-PCR; n = 6/group and each experiment was performed with 3 technical replicates. **(I–L)** Representative images and quantifications of EGFR, p38, p-p38, and CaMKII expressions in the brain; n = 3/group and each experiment was performed with 3 technical replicates. Except for **(D)**, the data are expressed as the mean ± SD. *p < 0.05 for the agomir-miR group versus the agomir-NC group. agomir-miR, VPA+ miR-30b-5p agomir; agomir-NC, VPA+ miR-30b-5p agomir NC.

We next tested whether miR-30b-5p is sufficient to reduce ASD-like behaviors in VPA-exposed rats. To test this hypothesis, we overexpressed the microRNA by intracerebroventricular infusion of a miR-30b-5p agomir in VPA-exposed rats. Interestingly, compared with VPA-exposed rats injected with agomir NC, VPA-exposed rats injected with the miR-30b-5p agomir significantly ameliorated ASD-like behaviors, including a greater sociability index (**Figure 6A**, p < 0.05) and social preference index (**Figure 6B**, p < 0.05), less time spent on self-grooming (**Figure 6C**, p < 0.05) and fewer buried marbles (**Figure 6D**, p < 0.05).

We further examined the effects of the miR-30b-5p agomir on neuroinflammation and EGFR expression. As shown in **Figures 6E–H**, miR-30b-5p was upregulated, while IL-6, TNF-α, and IL-1β were downregulated in the brains of VPA-exposed rats after injection of the agomir. In addition, Western blot analysis revealed that EGFR was decreased and was accompanied by a decrease in p-p38/p38 and CaMKII in the brains of VPA-exposed rats after injection of the agomir (**Figures 6I–L**, p < 0.05). Taken together, these findings suggest that the overexpression of miR-30b-5p ameliorates ASD-like behaviors by modulating EGFR through the MAPK signaling pathway and calcium signaling pathway in VPA-exposed rats.

## 4 Discussion

In this study, we characterized the exosome profiles in the plasma of a rat model of ASD. We found that the level of miR-30b-5p was significantly lower in the plasma exosomes of VPA-exposed rats than in those of sham rats. Plasma exosomal miR-30b-5p attenuated neuroinflammation by modulating EGFR through the MAPK signaling pathway and calcium signaling pathway. This study provides novel insights into the role of miR-30b-5p in plasma exosomes, which could be considered a potential therapeutic target for the treatment of ASD in the clinic.

Exosomal miRNAs, which have been validated as promising biomarkers for multiple diseases, have been shown to participate in central nervous system diseases (11–13). In this study, we observed that miR-30b-5p was significantly downregulated in the plasma exosomes of VPA-exposed rats, and the same result was found in the brain, which suggests that miRNAs in plasma exosomes might reflect changes in the brain (20). In addition, overexpression of miR-30b-5p attenuated ASD-like behaviors in VPA-exposed rats, which indicated that miR-30b-5p might have neuroprotective effects on VPA-exposed rats. miR-30b-5p reportedly has beneficial effects on attenuating apoptosis following epilepsy (24). However, whether plasma exosomal miR-30b-5p is involved in ASD has not been elucidated. As previous studies reported (5, 7), we found that the levels of inflammatory factors were increased in the brains of VPA-exposed rats. However, overexpressing miR-30b-5p reversed these results, which indicated that miR-30b-5p played a neuroprotective role by attenuating neuroinflammation in a rat model of ASD. So far, studies on the exosomal miR-30b-5p in people with ASD is lacking, our study is the first to report the dysregulation of exosomal miR-30b-5p in an ASD rat model. Specially, a recent study reported that miR-521, miR-501-5p, and miR-663b were significantly upregulated, while miR-222-5p was downregulated in peripheral blood exosomes of ASD children (30). It is worth noting that miR-501-5p, miR-663b, and miR-222-5p in people with ASD and miR-30b-5p in ASD rat model were all associated with inflammation (31–33), indicating a common pattern of “inflammatory miRNA dysregulation” in ASD across species. However, the protective mechanisms of miR-30b-5p in ASD need to be clarified.

Epidermal growth factor receptor (EGFR) is a transmembrane protein that belongs to the avian erythroblastosis B family (34). The activation of EGFR can stimulate the production of proinflammatory factors (35, 36). A previous study reported that EGFR plays an important role in mediating neuroinflammation in Alzheimer’s disease (37). In our study, we found that the levels of inflammatory factors and EGFR were increased in VPA-exposed rats. Russo et al. (38) found that plasma EGFR was higher in ASD children compared to healthy controls. The consistency of EGFR upregulation in both people with ASD and ASD rat model strongly suggests that the EGFR regulatory axis may be related to ASD. In addition, the expression level of EGFR is regulated by miRNAs (39–41). miR-133b-5p can target EGFR and aggravate inflammation in a rat model exposed to PM2.5 (39). However, whether miR-30b-5p can regulate the expression of EGFR in an ASD rat model is not clear. In our study, we found that the level of miR-30b-5p was decreased while the levels of inflammatory factors and EGFR were increased in VPA-exposed rats. In addition, miR-30b-5p overexpression reduced the expression of inflammatory factors and EGFR. In summary, we demonstrated that miR-30b-5p inhibited neuroinflammation by modulating EGFR.

In the present study, a pathway relationship network indicated that the MAPK signaling pathway and calcium signaling pathway were the most important pathways in VPA-exposed rats. The MAPK signaling pathway plays crucial roles in various cellular functions, including inflammation, the stress response, cell proliferation, and apoptosis (42). It has been reported that EGFR can trigger the MAPK signaling pathway (43–45). EGFR and its downstream MAPK signaling pathway are found increased in chronic obstructive pulmonary disease (COPD) (45). Intriguingly, vitamin E can alleviate inflammation in a COPD rat model by inhibiting the MAPK signaling pathway through decreasing EGFR expression (45). Furthermore, the MAPK signaling pathway, which influences the inflammatory process, can be regulated by exosomal miR-122-5p in autoimmune-mediated liver disease (46). We found that the levels of inflammatory factors, EGFR, and p-p38/p38 were increased in the brains of VPA-exposed rats and decreased with miR-30b-5p overexpression. Consistent with our data, previous studies reported that p-p38/p38 levels were significantly increased in the brains of VPA-exposed rats, and pharmacological inhibition of p-p38/p38 reduced neuroinflammation (47, 48). These findings suggest that the EGFR/MAPK signaling pathway is involved in miR-30b-5p-mediated neuroinflammation in an ASD rat model.

The calcium signaling pathway, another important pathway, has been reported to be associated with inflammation (49, 50). EGFR can mediate the intracellular calcium signaling pathway and regulate tumor growth in cholangiocarcinoma (51). Moreover, miR-152-3p inhibits neuroinflammation by modulating calcium signaling pathway in vascular dementia (52). However, whether miR-30b-5p inhibits inflammation in a rat model of ASD by modulating EGFR through calcium signaling pathway is unclear. In this study, we found CaMKII was increased in the brains of VPA-exposed rats. Consistent with our finding, Rhee et al. (53) reported increased CaMKII expression in the hippocampus of ASD mice model. The consistency of the upregulated CaMKII in ASD animal models suggests that CaMKII may be a therapeutic target. In addition, we found the levels of inflammatory factors, EGFR, and CaMKII were decreased with miR-30b-5p overexpression. These findings suggest that miR-30b-5p inhibits neuroinflammation by modulating the EGFR/calcium signaling pathway in a rat model of ASD.

## 5 Conclusion

In this study, plasma exosomal miR-30b-5p attenuated neuroinflammation in a rat model of ASD by modulating EGFR through the MAPK signaling pathway and calcium signaling pathway. This study provides novel perspectives on plasma exosomal miR-30b-5p, which could be considered a potential therapeutic target for the treatment of ASD in the clinic.

## Data Availability

The raw data supporting the conclusions of this article will be made available by the authors, without undue reservation.
